# Prevalence and prognosis of hyperdynamic left ventricular systolic function in septic patients: a systematic review and meta-analysis

**DOI:** 10.1186/s13613-024-01255-9

**Published:** 2024-02-03

**Authors:** Ryota Sato, Filippo Sanfilippo, Daisuke Hasegawa, Narut Prasitlumkum, Abhijit Duggal, Siddharth Dugar

**Affiliations:** 1 Division of Critical Care Medicine, Department of Medicine, The Queen’s Medical Center, Honolulu, HI USA; 2 Department of Anaesthesia and Intensive Care, A.O.U. Policlinico-San Marco, Site “Policlinico G. Rodolico”, Via S. Sofia N 78, 95123 Catania, Italy; 3 Department of Internal Medicine, Mount Sinai Beth Israel, New York, NY USA; 4 Department of Cardiovascular Medicine, Mayo Clinic, Rochester, MN USA; 5 Department of Critical Care Medicine, Respiratory Institute, Cleveland Clinic, 9500 Euclid Ave, Cleveland, OH 44195 USA; 6 Cleveland Clinic Lerner College of Medicine, Cleveland, OH USA

**Keywords:** Sepsis, Septic shock, Hyperkinetic, Hyperdynamic, Mortality, Left ventricular ejection fraction

## Abstract

**Purpose:**

The prevalence of hyperdynamic left ventricular (LV) systolic function in septic patients and its impact on mortality remain controversial. In this systematic review and meta-analysis, we investigated the prevalence and association of hyperdynamic LV systolic function with mortality in patients with sepsis.

**Methods:**

We searched MEDLINE, Cochrane Central Register of Controlled Trials, and Embase. Primary outcomes were the prevalence of hyperdynamic LV systolic function in adult septic patients and the associated short-term mortality as compared to normal LV systolic function. Hyperdynamic LV systolic function was defined using LV ejection fraction (LVEF) of 70% as cutoff. Secondary outcomes were heart rate, LV end-diastolic diameter (LVEDD), and E/e’ ratio.

**Results:**

Four studies were included, and the pooled prevalence of hyperdynamic LV systolic function was 18.2% ([95% confidence interval (CI) 12.5, 25.8]; *I*^*2*^ = 7.0%, *P* < 0.0001). Hyperdynamic LV systolic function was associated with higher mortality: odds ratio of 2.37 [95%CI 1.47, 3.80]; *I*^*2*^ = 79%, *P* < 0.01. No difference was found in E/e’ (*P* = 0.43) between normal and hyperdynamic LV systolic function, while higher values of heart rate (mean difference: 6.14 beats/min [95%CI 3.59, 8.69]; *I*^*2*^ = 51%, *P* < 0.0001) and LVEDD (mean difference: − 0.21 cm [95%CI − 0.33, − 0.09]; *I*^*2*^ = 73%, *P* < 0.001) were detected in patients with hyperdynamic LV systolic function.

**Conclusion:**

The prevalence of hyperdynamic LV systolic function is not negligible in septic patients. Such a finding is associated with significantly higher short-term mortality as compared to normal LV systolic function.

**Supplementary Information:**

The online version contains supplementary material available at 10.1186/s13613-024-01255-9.

## Introduction

Sepsis is a life-threatening condition related to infection characterized by dysregulated inflammatory response and organ failure. While sepsis is known to cause myocardial dysfunction [1], the definition of septic cardiomyopathy still represents a challenge because echocardiographic parameters are largely influenced by the variability of loading conditions and for several other reasons [2]. Since the original paper regarding sepsis-induced myocardial dysfunction by Parker et al., the majority of the studies have concentrated on the impact on patients’ outcomes of an impaired LV function using various definitions [3–5]. However, it should be noted that it is also common to encounter hyperdynamic LV systolic function in patients with sepsis. A study reported that hyperdynamic LV systolic function in patients with non-traumatic undifferentiated shock was highly specific to sepsis, though not very sensitive [6]. Hyperdynamic LV systolic function could be a normal response to under-resuscitation and vasoplegia, but once resuscitation and loading conditions have been optimized, sympathetic overstimulation from catecholamines and cytokines may play a role in generating hyperdynamic LV function in patients admitted to the intensive care unit (ICU) [5]. A recent study by our group in a large cohort of septic patients suggested that hyperdynamic LV function was associated with higher mortality even after adjusting for fluid status and vasopressor requirement [7]. However, despite an increasing number of studies dragging attention on hyperdynamic LV systolic function, limited information exists on its prevalence in sepsis and whether this condition is convincingly associated with higher mortality. To determine the prevalence and the clinical impact of hyperdynamic LV function in patients with sepsis, we conducted a systematic review and meta-analysis.

## Methods

### Protocol registration

Our systematic review and meta-analysis was conducted following the Preferred Reporting Items for Systematic Reviews and Meta-Analyses statement [8]. The protocol was submitted to the PROSPERO for the assessment (CDR42023409471).

### Search strategy

We used the following keywords: sepsis, septic shock, severe sepsis, hyperdynamic, hyperkinetic, left ventricular function, and systolic dysfunction for the systematic search on MEDLINE, the Cochrane Central Register of Controlled Trials, and Embase on 03/22/2023. The search strategy of each search engine is shown in Additional file 6: Table S1.

### Study selection and inclusion criteria

Two authors screened the abstracts and titles based on the following inclusion criteria. We then retrieved and reviewed the full texts.

The inclusion (PECOS) criteria were as follows:

1. Patient population: Adult (≥ 18 years old) patients with sepsis according to the recommendation at the time of study or International Classification of Diseases-9 or -10 coding.
2. Exposure: Hyperdynamic LV systolic function as defined by the authors. Of these, the definition by the LV ejection fraction (LVEF) cutoff of 70% [9] was used for the meta-analysis of the primary outcome.
3. Control: Septic patients with normal LV systolic function as defined by the authors.
4. Outcomes were divided into primary and secondary outcomes. Primary outcome: Short-term mortality (defined as < 90-day mortality, in-hospital mortality, or intensive care unit mortality depending on the availability). If more than one mortality timing was available, we selected the longest one. Secondary outcomes: Average E/e’ ratio, LV end-diastolic diameter (LVEDD), and heart rate and vasopressor dosage at the time of echocardiography
5. Study type: Randomized controlled trials, cohort studies, cross-sectional studies.

The exclusion criteria were as follows:

1. Conference proceedings.
2. Studies reported in a language other than the English language.
3. Studies that did not report the consecutive number of patients required to analyze the prevalence of hyperdynamic heart

In the case of any conflicts in regard to the inclusion or exclusion of studies, we discussed them in detail until a consensus was reached.

### Data extraction

The data were collected from included articles based on a standardized form on Microsoft Excel™, which included the following: author’s last name, publication year, country, sample size, study setting, study period, the definition of sepsis, definition of hyperdynamic LV systolic function, the timing of echocardiogram, and outcomes reported. For studies that did not report data required for the analyses, we queried authors for the necessary data.

### Quality assessment and grade of evidence

Two authors (R.S., D.H.) independently assessed the quality of the studies included in the primary meta-analysis using a modified version of the Newcastle–Ottawa quality assessment scale [10]. When there were different assessments, we discussed them in detail until consensus was achieved.

Grade of evidence was performed according to the recommendations of the Grading of Recommendations Assessment, Development, and Evaluation (GRADE) working group by two authors (R.S., F.S.) using the GRADEpro software [11].

### Statistical analysis

The meta-analyses were performed using the random-effects model. The pooled odds ratio (OR) for short-term mortality was reported as point estimates with 95% CIs and *P*-values. This OR was the risk of short-term mortality for the hyperdynamic group against the control group (normal LV systolic function). We reported the mean difference (MD) for the average E/e’ ratio, heart rate, and LVEDD. When data in the reference study were reported as the median and interquartile range (IQRs), we converted them to mean and standard deviation according to Wan et al. [12]. For the prevalence of hyperdynamic LV function, a double arcsine transformation was used to stabilize the variance for the pooled prevalence [13]. The pooled prevalence value was reported with 95% confidence intervals (CI) and *P*-value. We also performed several sensitivity analyses. The first one was conducted including studies that used different criteria than the LVEF cutoff of 70% to define hyperdynamic LV systolic function. The second sensitivity analysis was performed with the “leave one study out at a time” approach. The third sensitivity analysis was performed including studies that reported mortality of hyperdynamic LV systolic function in other ICU populations with variable admission diagnoses.

We examined statistical heterogeneity using the Chi-square test and the *I*^2^ statistic as the proportion of total variability explained by heterogeneity [14]. We predefined substantial heterogeneity as a *P*-value of < 0.10 with the Chi-square test or an *I*^2^ value of > 50%.

We generated funnel plots for the analysis of the short-term mortality in which we plotted the log ORs against their standard errors and tested the symmetry of the funnel plots using both Begg’s rank correlation test and Egger’s linear regression test. When the publication bias was detected, we planned to perform the trim-and-fill method to fill the missing data to modify the publication bias and report the point estimate with CI with missing data filled [15, 16]. All statistical analyses were performed using Review Manager Version 5.4. (RevMan; The Cochrane Collaboration 2012, The Nordic Cochrane Center, Copenhagen, Denmark) and Comprehensive Meta-analysis version 3 software (Biostat Inc., Eaglewood, NJ, USA). *P* < 0.05 was considered statistically significant.

We also conducted a trial sequential analysis (TSA) to evaluate the robustness of the findings of the primary outcome. We used the freely available TSA Software (Copenhagen Trial Unit’s TSA Software®; Copenhagen, Denmark). The estimated effect was computed using the average result from the forest plot of the meta-analysis. The information size and the adjusted boundaries were computed assuming an alpha risk of 5% and a beta risk of 10% (restrictive). Further details on TSA and its interpretation are available elsewhere [17].

## Results

### Literature search

Our systematic search identified 1306 articles. After duplicates were removed, 1253 titles and abstracts were screened and 77 articles were reviewed in detail. Of these articles, 67 were excluded (different outcomes reported, *n* = 42; different population, *n* = 23; conference proceedings, *n* = 2). One additional study was found by manual search and was not listed in any of the databases that we used for the systematic search [18]. However, this study was only included in the sensitivity analysis as this study used a definition other than the LVEF cutoff of 70% for hyperdynamic LV systolic function.

**Fig. 1:**
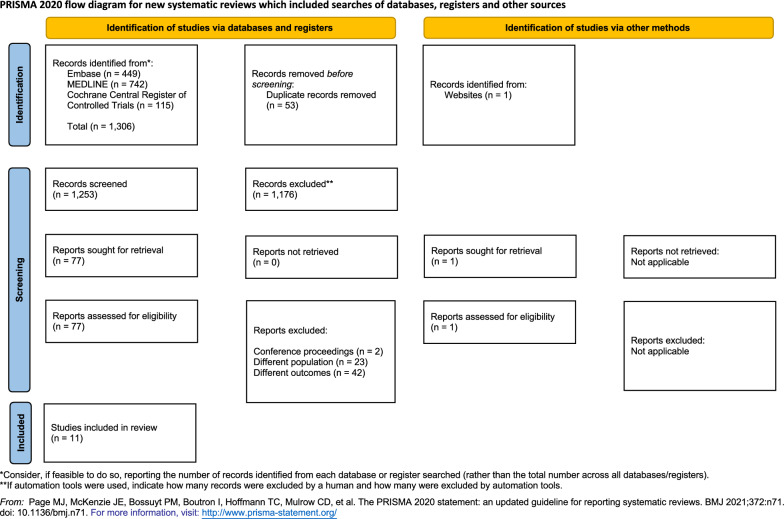
Preferred Reporting Items for Systematic Reviews and Meta-Analyses (PRISMA) 2020 chart

Eleven studies were included in this systematic review. Of these, four studies with a total of 3427 patients were included in the meta-analysis for the primary outcome [7, 19–21]. Two of these defined hyperdynamic LV systolic function as LVEF > 70% and two others used LVEF ≥ 70%. The PRISMA flowchart of the study selection is shown in Fig. 1. The characteristics of the studies are summarized in Table 1. The sample size of studies included in the primary analysis ranged from 100 to 2145 patients. For the analysis of the pooled prevalence, we used the entire consecutive population of sepsis (not only hyperdynamic vs. normal LV systolic function but also reduced LV systolic function), which ranged from 111 to 3151 patients. Outcomes were summarized in Additional file 7: Table S2.

**Table 1:** Characteristics of each study

Authors	Country	Sample size	Setting	Study period	Definition of sepsis	Definition of hyperdynamic state	Timing of echocardiogram	Definition of short-term outcome
Dugar 2023	United States	3151	Retrospective single-center observational	January 2011–December 2020	Sepsis-3	LVEF ≥ 70%	Within 3 days of MICU admission	In-hospital mortality
Chotalia 2022	United Kingdom	1014	Retrospective single-center observational	April 2016–December 2019	Sepsis-3	LVEF > 70%	Within 7 days of sepsis	90-day mortality
Shin 2020	Korea	366	Retrospective single-center observational	November 2016–December 2018	Sepsis-3	LVEF ≥ 70%	Within 48 h of sepsis	In-hospital mortality
Chang 2015	Taiwan	111	Prospective two-center observational	January 2011–July 2013	Sepsis-2	LVEF > 70%	Within 24 h of ICU admission	In-hospital mortality

Seven studies used different definitions of hyperdynamic LV systolic function other than the LVEF cutoff of 70%. These seven studies were by Boissier et al. (hyperdynamic LV systolic function definition was LVEF > 60%) [22], Geri et al. (definition based on hierarchical clustering) [23], Havaldar (definition by visual gestalt) [24], Weng et al. and Zaytoun et al. (tissue-Doppler velocity S’ ≥ 9 cm/m^2^) [18, 25], Vieillard-Baron et al. (definition relying on cardiac index > 4L/min^2^) [26], and Baumgartner et al. (definition relying on cardiac index > 7L/min^2^)[27]. Among these, Geri et al. included a subset of septic patients from two previous multicenter prospective observational studies [28, 29] to perform the hierarchical clustering [23]. In this study, we regarded that this study included consecutive patients with sepsis. These studies were included in a sensitivity analysis.

### Primary outcomes

**Fig. 2:**
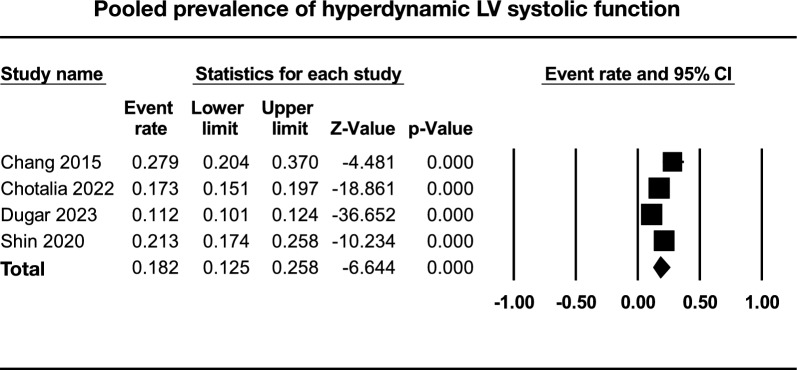
Forest plot of the pooled prevalence of hyperdynamic left ventricular (LV) systolic function. *CI* confidence interval

**Fig. 3:**
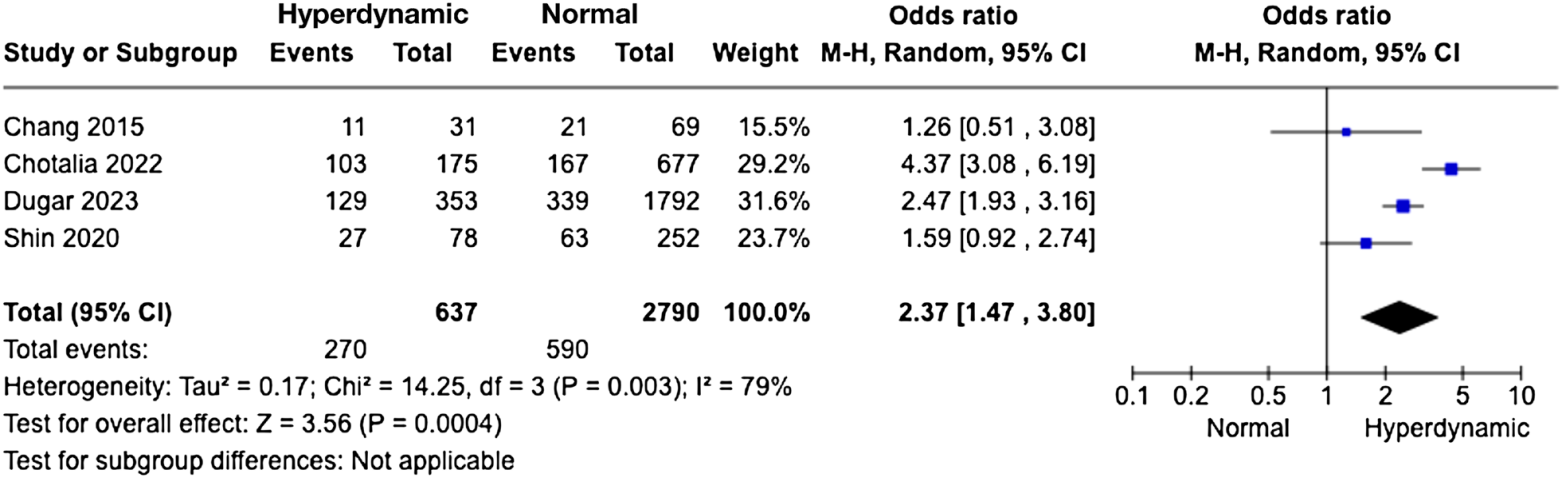
Forest plots of the short-term mortality in patients with hyperdynamic left ventricular (LV) systolic function as compared to normal LV systolic function. *CI* confidence interval

Four studies with a total of 3427 patients with sepsis reported the prevalence of hyperdynamic LV systolic function. The pooled prevalence was 18.2% (95%CI 12.5, 25.8; *I*^*2*^ = 7.0%, *P* < 0.0001) (Fig. 2). All the four studies reported short-term mortality (Fig. 3) which was significantly higher for hyperdynamic LV function as compared to normal LV systolic function, with an OR of 2.37 (95%CI 1.47, 3.80; *I*^*2*^ = 79%; *P* < 0.01).

### Secondary outcomes

**Fig. 4:**
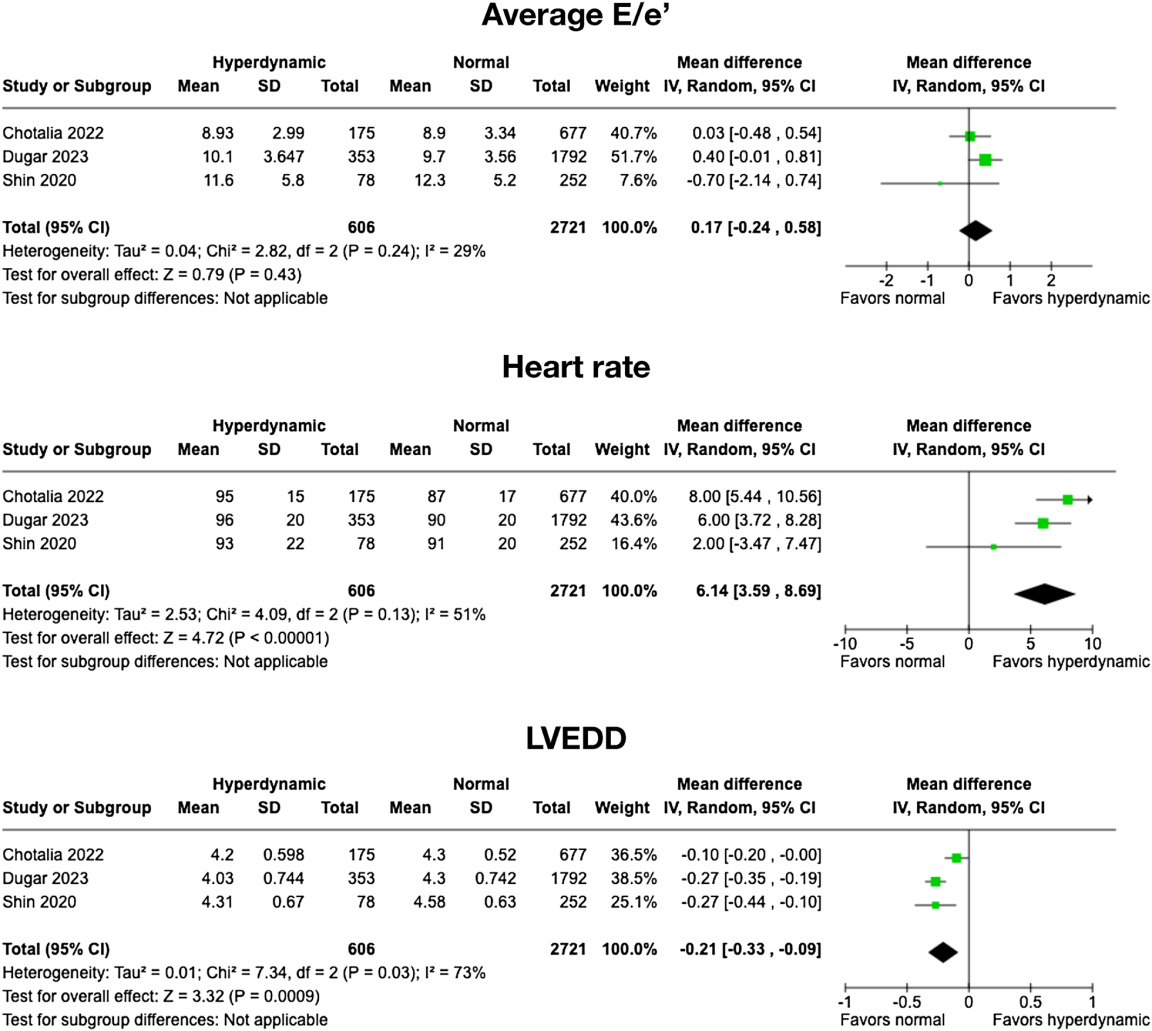
Forest plots of the three secondary outcomes comparing patients with hyperdynamic left ventricular (LV) systolic function with normal LV systolic function. From top to bottom: average E/e’, heart rate, and LV end-diastolic diameter (LVEDD). *CI* confidence interval

Three studies were included in the rest of the analyses for the secondary outcomes [7, 19, 20]. The average E/e’ ratio was not significantly different between hyperdynamic and normal LV systolic function [mean difference (MD), 0.17; (95%CI − 0.24, 0.58); *I*^2^ = 29%; *P* = 0.43]. As compared to patients with normal LV systolic function, those with hyperdynamic LV systolic function had higher heart rates with MD 6.14 beats/min (95%CI 3.59, 8.69); *I*^2^ = 51%, *P* < 0.0001 and smaller LVEDD with MD − 0.21 cm (95%CI − 0.33, − 0.09); *I*^2^ = 73%; *P* < 0.001. These results are shown in Fig. 4. As only two studies reported vasopressor dosage at the time of echocardiography [7, 19], we decided not to perform a meta-analysis of this secondary outcome.

### Sensitivity analysis

A sensitivity analysis was performed by adding the results of the seven articles, which were not included in the main analysis due to diverse definitions of hyperdynamic heart [18, 22–27]. We found a pooled OR of short-term mortality associated with hyperdynamic LV systolic function of 2.58 (95%CI 1.69, 3.95); *I*^*2*^ = 70%; *P* < 0.0001, Additional file 1: Fig. S1.

We also performed the sensitivity analysis including two studies assessing the impact of hyperdynamic LV systolic function in general critically ill patients and patients [9] with coronavirus disease 2019 [30]. In this sensitivity analysis, the adjusted OR was used in four studies [7, 9, 19, 30] and the unadjusted OR was used in the rest of two studies [20, 21]. We found a pooled OR of short-term mortality associated with hyperdynamic LV systolic function of 1.76 (95%CI 1.30, 2.37); *I*^*2*^ = 57.3%; *P* < 0.039, Additional file 2: Fig. S2.

In addition, we also conducted “leave-one-out at a time” analyses, thus excluding one of the four studies included in the primary analysis at a time. Except for the analysis excluding the largest study by Dugar et al., hyperdynamic LV systolic function remained significantly associated with higher odds of short-term mortality. Even in the analysis excluding Dugar et al., hyperdynamic LV systolic function tended to be associated with higher odds of short-term mortality (*P* = 0.07), supporting the findings of the primary analysis (Additional file 3: Fig. S3).

### Quality assessment and GRADE of evidence

All the included studies were deemed at low risk of bias, scoring 7 points on the Newcastle–Ottawa quality assessment scale. Details of the methodological assessment are shown in Additional file 8: Table S3.

The assessment of the grade of evidence is shown in Additional file 9: Table S4, and all the outcomes investigated scored as low overall certainty of evidence due to serious imprecision and the observational nature of the included studies.

### Publication bias

The funnel plots for the studies included in the primary analyses are shown in Additional file 4: Figure S4. Visual assessment does not suggest publication bias, and this was also confirmed using Begg’s rank correlation test (*P* = 0.734) and Egger’s linear regression test (*P* = 0.196).

### Trial sequential analysis

The TSA performed to evaluate the primary outcome of short-term mortality showed the robustness of the findings, with a Z-curve that crossed boundaries of significance; moreover, the number of patients included in the meta-analysis (*n* = 3427) was by far higher than the information size needed (*n* = 1331 patients) (Additional file 5: Fig. S5).

## Discussion

To the best of our knowledge, our meta-analysis represents the first attempt to unify the existing evidence for hyperdynamic LV systolic function in sepsis. In this systematic review and meta-analysis, we found a prevalence of hyperdynamic LV systolic function in sepsis of 18.2%, and such an echocardiographic finding was associated with a significantly higher risk of short-term mortality compared to normal LV systolic function with an OR of 2.37. Given that sepsis is the most frequent cause of admission to the ICU and the leading cause of death in hospitalized patients [31], an overall prevalence of around one in five patients is certainly not negligible. Hence, the diagnosis of hyperdynamic LV systolic function should probably trigger attention in the treating clinicians.

Our study is clinically important for two reasons. First, it describes the prevalence of hyperdynamic LV systolic function in the context of sepsis and its association with short-term mortality. In cohorts of critically ill patients [9] and in hospitalized coronavirus disease 2019 patients [30], hyperdynamic LV systolic function defined by LVEF > 70% was associated with significantly higher short-term mortality. This significant association between hyperdynamic LV systolic function and mortality, regardless of whether an underlying condition is sepsis or not, may suggest that hyperdynamic LV systolic function is a pathogenic condition requiring clinical attention.

Second, this study attempts to elucidate features of hyperdynamic LV systolic function in the context of sepsis. Unfortunately, other echocardiographic and hemodynamic parameters were inconstantly reported in the included studies. Notably, we tried to delineate the cardiovascular features of the hyperdynamic LV systolic function. For the secondary outcomes, we explored cardiovascular features of the hyperdynamic LV systolic function and found no differences in E/e’ ratio, suggesting that LV filling pressures (and possibly LV diastolic function) might not have been very different between groups. This is important as LV diastolic dysfunction has been associated with worse outcomes in critically ill patients [32–35]. Of note, E/e’ ratio correlates with left atrial pressure in critically ill patients [36, 37], and it is included by the recent guidelines among the parameters for the diagnosis and grading of LV diastolic dysfunction [38]. E/e’ ratio is also associated with prognosis in critically ill patients [33, 39]. As we compared hyperdynamic and normal LV systolic function, the similar value of E/e’ ratio between groups suggests that significant differences in left atrial pressures and/or LV diastolic function were unlikely. Conversely, we detected a significantly higher heart rate and smaller LVEDD in patients with hyperdynamic LV systolic function. These results may partially suggest greater sympathetic stimulation (tachycardia) due to concomitant reduction in preload (smaller LVEDD). However, it should be noted that the mean differences in heart rate and LVEDD between both groups were only ~ 6 beats/min and ~ 0.2 cm, suggesting the uncertain clinical meaningfulness of these findings. Even though the reason triggering LV hyperdynamic systolic function should be sought, given the higher mortality associated with hyperdynamic LV function in sepsis, clinicians should consider careful modifications of their hemodynamic management once observing this echocardiographic profile. For instance, a recent study by our group suggests that higher LVEF may have a favorable response to vasopressin with a greater reduction in norepinephrine dose and lower short-term mortality [40]. If tachycardia is recognized as “compensatory,” clinicians may try to restore the best conditions to reduce the chronotropic cardiac compensation. While Dugar et al. and Chotalia et al. reported that the fluid balance on the day of echocardiography was similar between hyperdynamic and normal LV systolic function groups [7, 19], clinicians need to ensure that volume status is optimized. Conversely, a non-compensatory form of tachycardia with concomitant hyperdynamic LV systolic function due to sympathetic overstimulation may benefit from ultra-short-acting beta-blockers [41]. Morelli et al. suggested that the difference in systolic and dicrotic pressure on arterial waveform may differentiate between responders and non-responders to beta-blockers, differentiating between compensatory and maladaptive (sympathetic overstimulation) origin of tachycardia [42]. Another option could be the use of alpha-2 receptor agonists (dexmedetomidine) to counteract the alpha-receptor downregulation [43], thereby positively modulating vascular responsiveness to norepinephrine [44]. As such, this hemodynamic phenotype should require more attention, and further studies regarding effective therapeutic intervention are warranted.

## Limitations

While our study has strengths, there are several limitations that should be acknowledged. First, we included all single-center observational studies, hence there could be some issues of selection bias and uncertainties regarding generalizability and external validity. On the contrary, we ensured that all included studies enrolled consecutive patients to calculate the pooled prevalence of hyperdynamic LV systolic function in patients with sepsis, minimizing such risk. Second, we used an arbitrary cutoff to define hyperdynamic LV systolic function as an accepted definition is still lacking. However, the sensitivity analyses including other studies adopting different definitions of hyperdynamic LV systolic function supported the validity of our primary result, with an almost identical OR. In a recent study, we described the linear relationship between tissue-Doppler-derived LV systolic velocity and in-hospital mortality, also supporting our results [45]. Third, the sample size of the included studies was highly variable, ranging from 100 to over 3000 septic patients. This likely influenced the analysis with high statistical heterogeneity in the short-term mortality analysis (*I*^2^ = 79%). Fourth, the significant heterogeneity observed in this study requires a cautious interpretation. There were variations in the outcome measured (in-hospital mortality and 90-day mortality) and in the definition of sepsis (sepsis-2 vs. sepsis-3), both of which might also have contributed to high statistical heterogeneity. Prior studies have shown that the application of sepsis-3 as compared to sepsis-2 identifies fewer patients with septic shock; however, the mortality of those with septic shock based on sepsis-3 is higher as compared to septic shock based on sepsis-2 [46, 47]. In addition, both Dugar et al. and Chotalia et al. had a higher proportion of hyperdynamic LV systolic function in the septic shock group [7, 19]. Given the observational nature of these studies, it is difficult to discern whether hyperdynamic LV systolic function is a precursor or outcome of higher severity of sepsis or vasopressor usage. On the contrary, the results remained significant in most of the sensitivity analyses and the TSA supported the robustness of our findings. Therefore, the association between hyperdynamic LV systolic function and higher mortality appeared to be robust. Lastly, there was a variation in the timing of echocardiography, ranging from within 24 h to 7 days of admission to the ICU or sepsis onset. This could affect the phase of sepsis where the echocardiogram was performed and hence, the prevalence of observed hyperdynamic LV function and interpretation of the secondary analyses. As E/e’ and LVEDD can change through clinical course, the data included in these secondary analyses may not necessarily represent the association between hyperdynamic LV systolic function and volume status as the E/e’ or LVEDD are non-invasive measures of volume status at a particular phase in sepsis. On the contrary, as shown in the sensitivity analysis, hyperdynamic LV systolic function was still associated with a significantly higher risk of short-term mortality despite various definitions of hyperdynamic LV systolic function and various timings of echocardiography.

## Conclusion

In conclusion, we found a prevalence of hyperdynamic LV function in sepsis of 18.2%, and this finding was associated with a significantly higher risk of short-term mortality. We found similar average E/e’ ratios between hyperdynamic and normal LV systolic function, suggesting that LV filling pressure may have had not a major role. Conversely, we detected a significant though clinically modest difference in heart rate and LVEDD. While more research is needed to understand and to differentiate the contribution of each underlying condition (hypovolemia, vasoplegia, and sympathetic overstimulation) in driving the hyperdynamic LV systolic function, clinicians should pay attention to these patients as they clearly seem to be at higher risk of mortality. Such patients may benefit from highly personalized hemodynamic management.

### Supplementary Information

**Additional file 1: Figure S1.** Sensitivity analysis including various definitions of hyperdynamic LV systolic function.

**Additional file 2: Figure S2.** Sensitivity analysis including Paonessa et al. and Rahman et al.

**Additional file 3: Figure S3.** Sensitivity analyses of short-term mortality excluding each study from four included studies.

**Additional file 4. **Funnel plot of included studies.

**Additional file 5. **Trial sequential analysis.

**Additional file 6: Table S1.** Search strategy.

**Additional file 7: Table S2.** Prevalence, heart rate, echocardiographic variables and Outcomes.

**Additional file 8: Table S3.** Newcastle–Ottawa Scale assessment of pooled studies.

**Additional file 9: Table S4.** GRADE (Grading of Recommendations, Assessment, Development, and Evaluations).

## Data Availability

The datasets used and analyzed during the current study are available from the corresponding author on reasonable request.

## Abbreviations

LV: Left ventricle/left ventricular
EF: Ejection fraction
TTE: Transthoracic echocardiography
PRISMA: Preferred Reporting Items for Systematic Reviews and Meta-Analyses
OR: Odds ratio
CIs: Confidence intervals
ICU: Intensive care unit
