# Supplementary material for: Prevalence and prognosis of hyperdynamic left ventricular systolic function in septic patients: a systematic review and meta-analysis
Source: Ann Intensive Care. 2024 Feb 3;14:22. doi: 10.1186/s13613-024-01255-9 (PMC10838258; doi:10.1186/s13613-024-01255-9)

Supplemental Figure S1

Sensitivity analysis including various definitions of hyperdynamic LV systolic function

Study or Subgroup	Hyperdynamic Events	Hyperdynamic Total	Normal Events	Normal Total	Weight	Odds ratio M-H, Random, 95% CI
**1.2.1 LVEF ≥70%**						
Chang 2015	11	31	21	69	10.0%	1.26 [0.51 , 3.08]
Chotalia 2022	103	175	167	677	16.2%	4.37 [3.08 , 6.19]
Dugar 2023	129	353	339	1792	17.1%	2.47 [1.93 , 3.16]
Shin 2020	27	78	63	252	13.9%	1.59 [0.92 , 2.74]
**Subtotal (95% CI)**		**637**		**2790**	**57.2%**	**2.37 [1.47 , 3.80]**
Total events:	270		590			
Heterogeneity: Tau² = 0.17; Chi² = 14.25, df = 3 (P = 0.003); I² = 79%						
Test for overall effect: Z = 3.56 (P = 0.0004)						
**1.2.2 S' ≥ 9cm/sec**						
Weng 2012	18	24	6	37	6.9%	15.50 [4.34 , 55.30]
Zaytoun 2020	15	45	5	15	7.1%	1.00 [0.29 , 3.45]
**Subtotal (95% CI)**		**69**		**52**	**14.0%**	**3.92 [0.27 , 57.55]**
Total events:	33		11			
Heterogeneity: Tau² = 3.35; Chi² = 9.15, df = 1 (P = 0.002); I² = 89%						
Test for overall effect: Z = 1.00 (P = 0.32)						
**1.2.3 LVEF ≥60%**						
Boissier 2017	30	48	35	84	11.8%	2.33 [1.13 , 4.83]
**Subtotal (95% CI)**		**48**		**84**	**11.8%**	**2.33 [1.13 , 4.83]**
Total events:	30		35			
Heterogeneity: Not applicable						
Test for overall effect: Z = 2.28 (P = 0.02)						
**1.2.4 Hierarchical clustering**						
Geri 2020	20	84	13	61	11.1%	1.15 [0.52 , 2.55]
**Subtotal (95% CI)**		**84**		**61**	**11.1%**	**1.15 [0.52 , 2.55]**
Total events:	20		13			
Heterogeneity: Not applicable						
Test for overall effect: Z = 0.35 (P = 0.72)						
**1.2.5 Visual gestalt**						
Havaldar 2018	2	5	1	11	2.1%	6.67 [0.44 , 101.73]
**Subtotal (95% CI)**		**5**		**11**	**2.1%**	**6.67 [0.44 , 101.73]**
Total events:	2		1			
Heterogeneity: Not applicable						
Test for overall effect: Z = 1.36 (P = 0.17)						
**1.2.6 Cardiac index ≥ 4L/min/m2**						
Vieillard-Baron 2001	6	6	6	27	1.8%	43.00 [2.13 , 869.97]
**Subtotal (95% CI)**		**6**		**27**	**1.8%**	**43.00 [2.13 , 869.97]**
Total events:	6		6			
Heterogeneity: Not applicable						
Test for overall effect: Z = 2.45 (P = 0.01)						
**1.2.7 Cardiac index ≥ 7L/min/m2**						
Baumgartner 1984	5	5	11	31	1.8%	19.61 [0.99 , 387.50]
**Subtotal (95% CI)**		**5**		**31**	**1.8%**	**19.61 [0.99 , 387.50]**
Total events:	5		11			
Heterogeneity: Not applicable						
Test for overall effect: Z = 1.95 (P = 0.05)						
**Total (95% CI)**		**854**		**3056**	**100.0%**	**2.58 [1.69 , 3.95]**
Total events:	366		667			
Heterogeneity: Tau² = 0.26; Chi² = 33.89, df = 10 (P = 0.0002); I² = 70%						
Test for overall effect: Z = 4.37 (P < 0.0001)						
Test for subgroup differences: Chi² = 9.28, df = 6 (P = 0.16), I² = 35.3%						

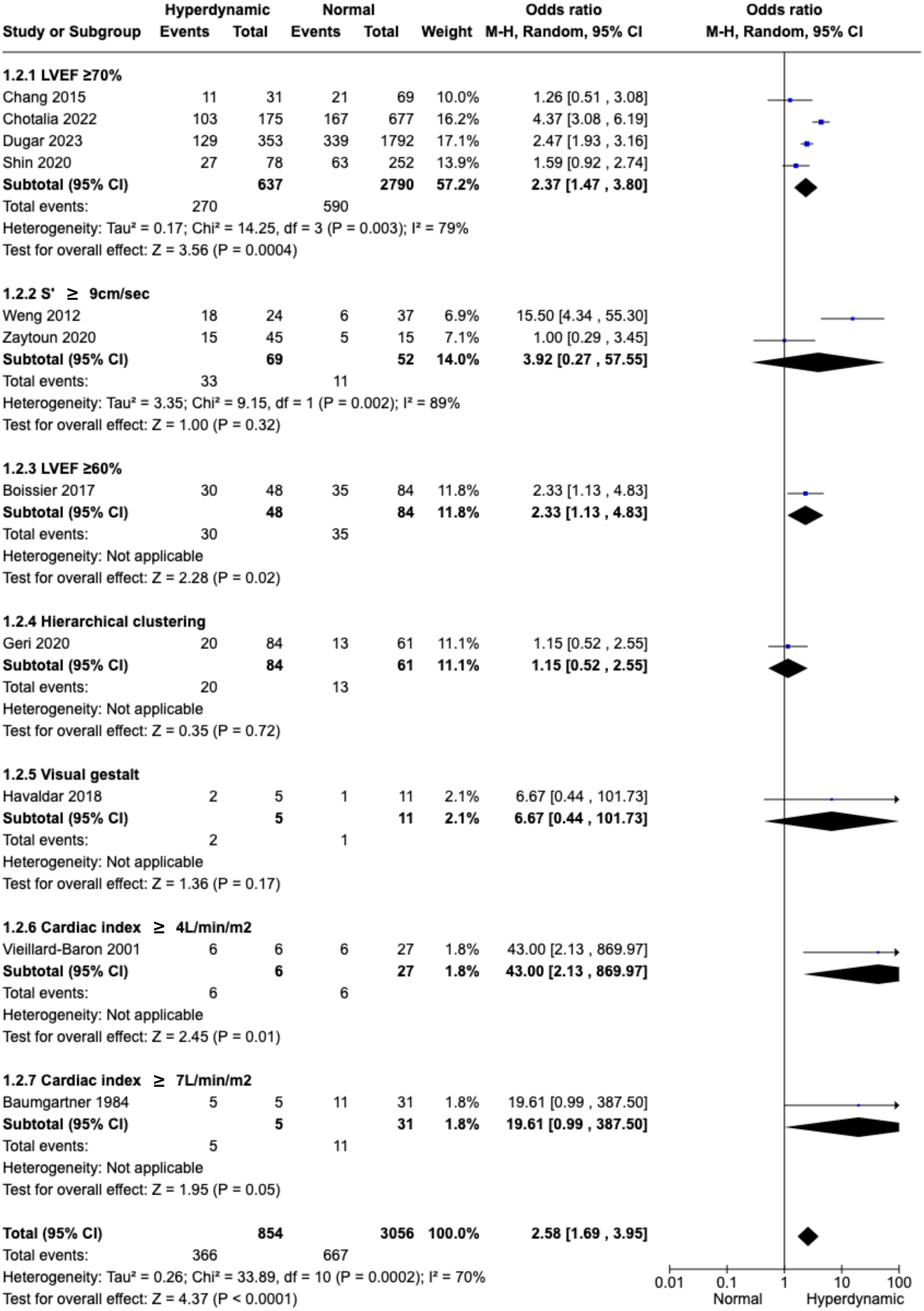

Supplement: Supplementary file 1 — Additional file 1: Figure S1. Sensitivity analysis including various definitions of hyperdynamic LV systolic function. [file 13613_2024_1255_MOESM1_ESM.pdf]
