# Supplementary material for: Prevalence and prognosis of hyperdynamic left ventricular systolic function in septic patients: a systematic review and meta-analysis
Source: Ann Intensive Care. 2024 Feb 3;14:22. doi: 10.1186/s13613-024-01255-9 (PMC10838258; doi:10.1186/s13613-024-01255-9)

Supplemental Figure S2
Sensitivity analysis including Paonessa et al. and Rahman et al.

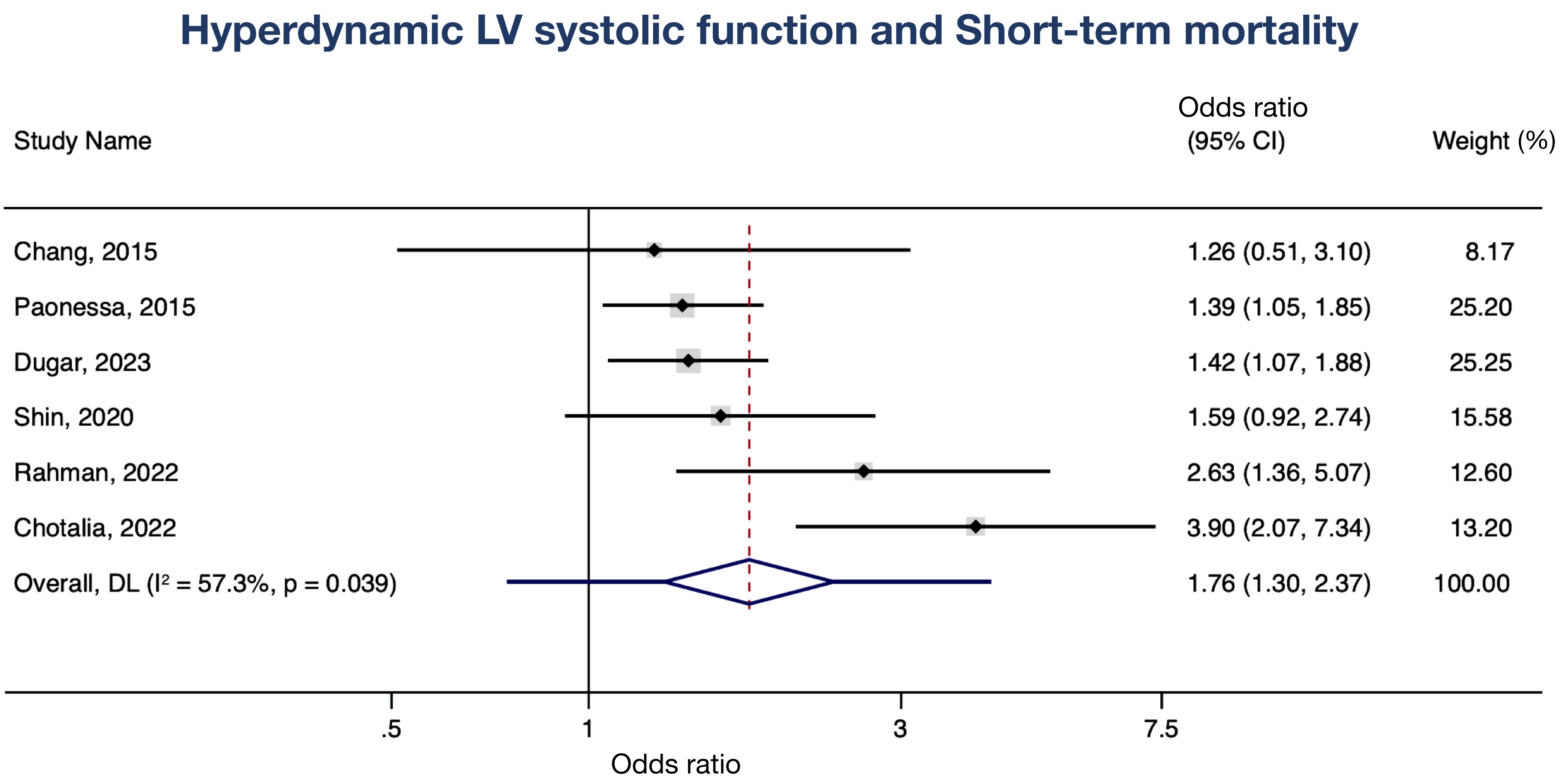

Supplement: Supplementary file 2 — Additional file 2: Figure S2. Sensitivity analysis including Paonessa et al. and Rahman et al. [file 13613_2024_1255_MOESM2_ESM.pdf]
