# Supplementary material for: Prevalence and prognosis of hyperdynamic left ventricular systolic function in septic patients: a systematic review and meta-analysis
Source: Ann Intensive Care. 2024 Feb 3;14:22. doi: 10.1186/s13613-024-01255-9 (PMC10838258; doi:10.1186/s13613-024-01255-9)

Supplemental Figure S3

Sensitivity analyses of short-term mortality excluding each study from 4 included studies

Sensitivity analysis without Dugar et al.

Study or Subgroup	Experimental Events	Experimental Total	Control Events	Control Total	Weight	Odds ratio M-H, Random, 95% CI
✔ Chang 2015	11	31	21	69	27.6%	1.26 [0.51 , 3.08]
✔ Chotalia 2022	103	175	167	677	37.8%	4.37 [3.08 , 6.19]
✖ Dugar 2023	129	353	339	1792	0.0%	2.47 [1.93 , 3.16]
✔ Shin 2020	27	78	63	252	34.5%	1.59 [0.92 , 2.74]
**Total (95% CI)**		**284**		**998**	**100.0%**	**2.18 [0.95 , 5.04]**
Total events:	141		251			

Heterogeneity: $Tau^2 = 0.45$; $Chi^2 = 13.29$, df = 2 (P = 0.001); $I^2 = 85\%$

Test for overall effect: Z = 1.83 (P = 0.07)

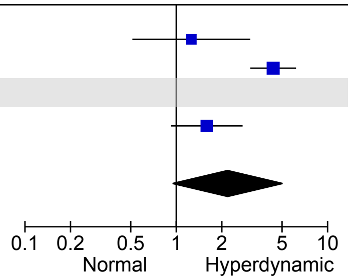

Sensitivity analysis without Chotalia et al.

Study or Subgroup	Experimental Events	Experimental Total	Control Events	Control Total	Weight	Odds ratio M-H, Random, 95% CI
✔ Chang 2015	11	31	21	69	15.2%	1.26 [0.51 , 3.08]
✖ Chotalia 2022	103	175	167	677	0.0%	4.37 [3.08 , 6.19]
✔ Dugar 2023	129	353	339	1792	54.9%	2.47 [1.93 , 3.16]
✔ Shin 2020	27	78	63	252	29.9%	1.59 [0.92 , 2.74]
**Total (95% CI)**		**462**		**2113**	**100.0%**	**1.95 [1.31 , 2.90]**
Total events:	167		423			

Heterogeneity: $Tau^2 = 0.06$; $Chi^2 = 3.70$, df = 2 (P = 0.16); $I^2 = 46\%$

Test for overall effect: Z = 3.31 (P = 0.0009)

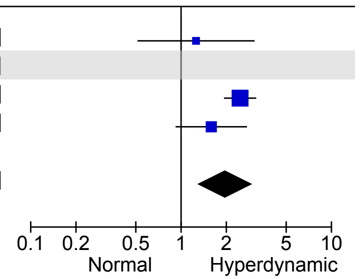

Sensitivity analysis without Shin et al.

Study or Subgroup	Experimental Events	Experimental Total	Control Events	Control Total	Weight	Odds ratio M-H, Random, 95% CI
✔ Chang 2015	11	31	21	69	20.2%	1.26 [0.51 , 3.08]
✔ Chotalia 2022	103	175	167	677	38.3%	4.37 [3.08 , 6.19]
✔ Dugar 2023	129	353	339	1792	41.5%	2.47 [1.93 , 3.16]
✖ Shin 2020	27	78	63	252	0.0%	1.59 [0.92 , 2.74]
**Total (95% CI)**		**559**		**2538**	**100.0%**	**2.68 [1.56 , 4.60]**
Total events:	243		527			

Heterogeneity: $Tau^2 = 0.17$; $Chi^2 = 10.28$, df = 2 (P = 0.006); $I^2 = 81\%$

Test for overall effect: Z = 3.57 (P = 0.0004)

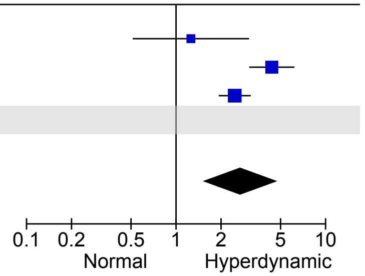

Sensitivity analysis without Chang et al.

Study or Subgroup	Experimental Events	Experimental Total	Control Events	Control Total	Weight	Odds ratio M-H, Random, 95% CI
✖ Chang 2015	11	31	21	69	0.0%	1.26 [0.51 , 3.08]
✔ Chotalia 2022	103	175	167	677	34.6%	4.37 [3.08 , 6.19]
✔ Dugar 2023	129	353	339	1792	37.7%	2.47 [1.93 , 3.16]
✔ Shin 2020	27	78	63	252	27.8%	1.59 [0.92 , 2.74]
**Total (95% CI)**		**606**		**2721**	**100.0%**	**2.66 [1.61 , 4.39]**
Total events:	259		569			

Heterogeneity: $Tau^2 = 0.16$; $Chi^2 = 11.40$, df = 2 (P = 0.003); $I^2 = 82\%$

Test for overall effect: Z = 3.83 (P = 0.0001)

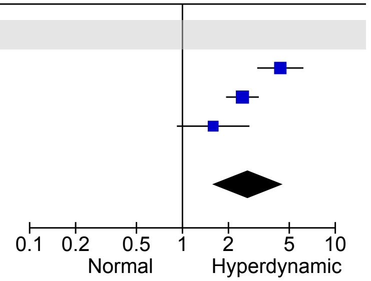

Supplement: Supplementary file 3 — Additional file 3: Figure S3. Sensitivity analyses of short-term mortality excluding each study from four included studies. [file 13613_2024_1255_MOESM3_ESM.pdf]
