# Supplementary figures and images for: Prevalence and prognosis of hyperdynamic left ventricular systolic function in septic patients: a systematic review and meta-analysis
Source: Ann Intensive Care. 2024 Feb 3;14:22. doi: 10.1186/s13613-024-01255-9 (PMC10838258; doi:10.1186/s13613-024-01255-9)

Supplemental Figure S4

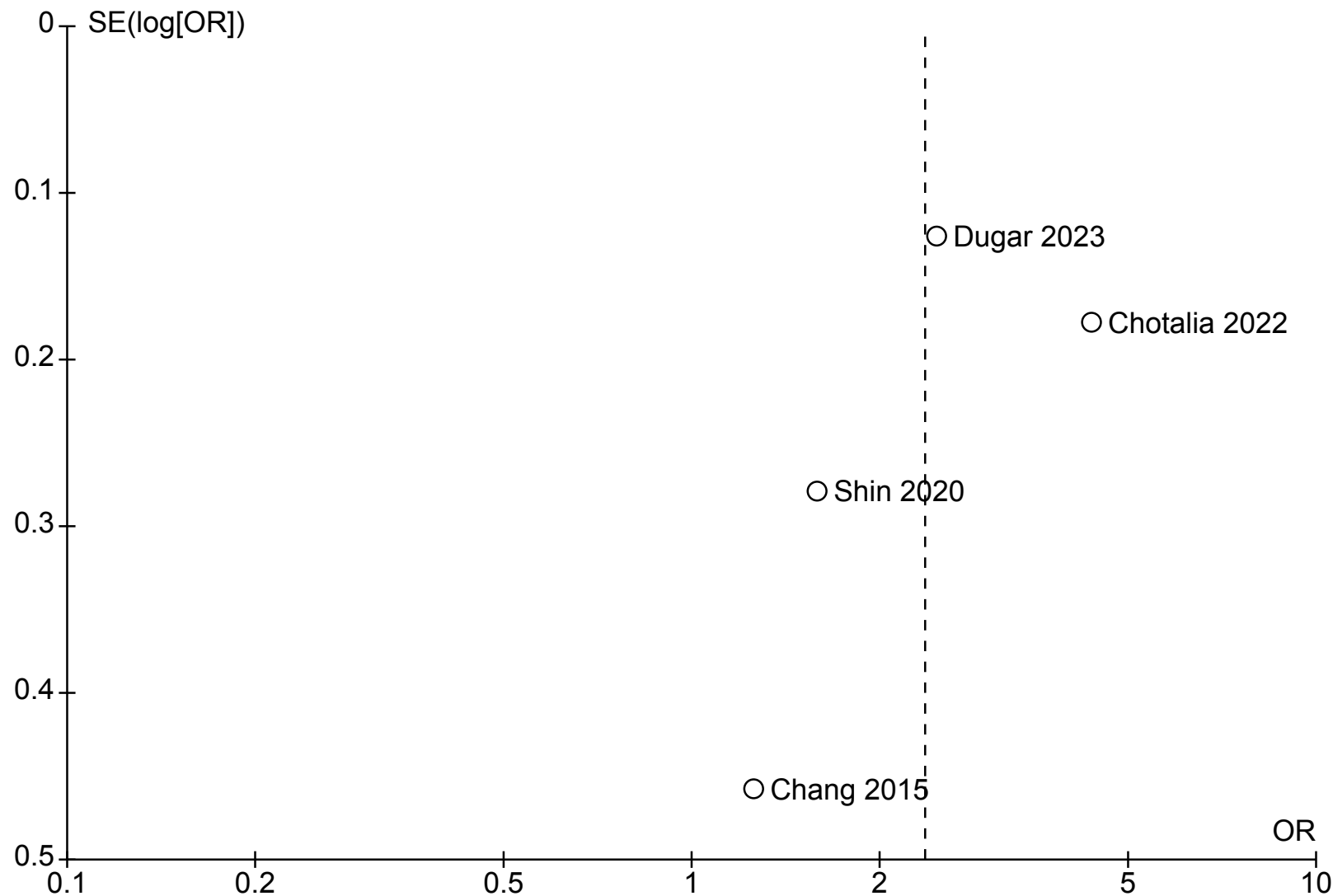

Supplement: Supplementary file 4 — Additional file 4. Funnel plot of included studies. [file 13613_2024_1255_MOESM4_ESM.pdf]

## Supplemental Figure S5

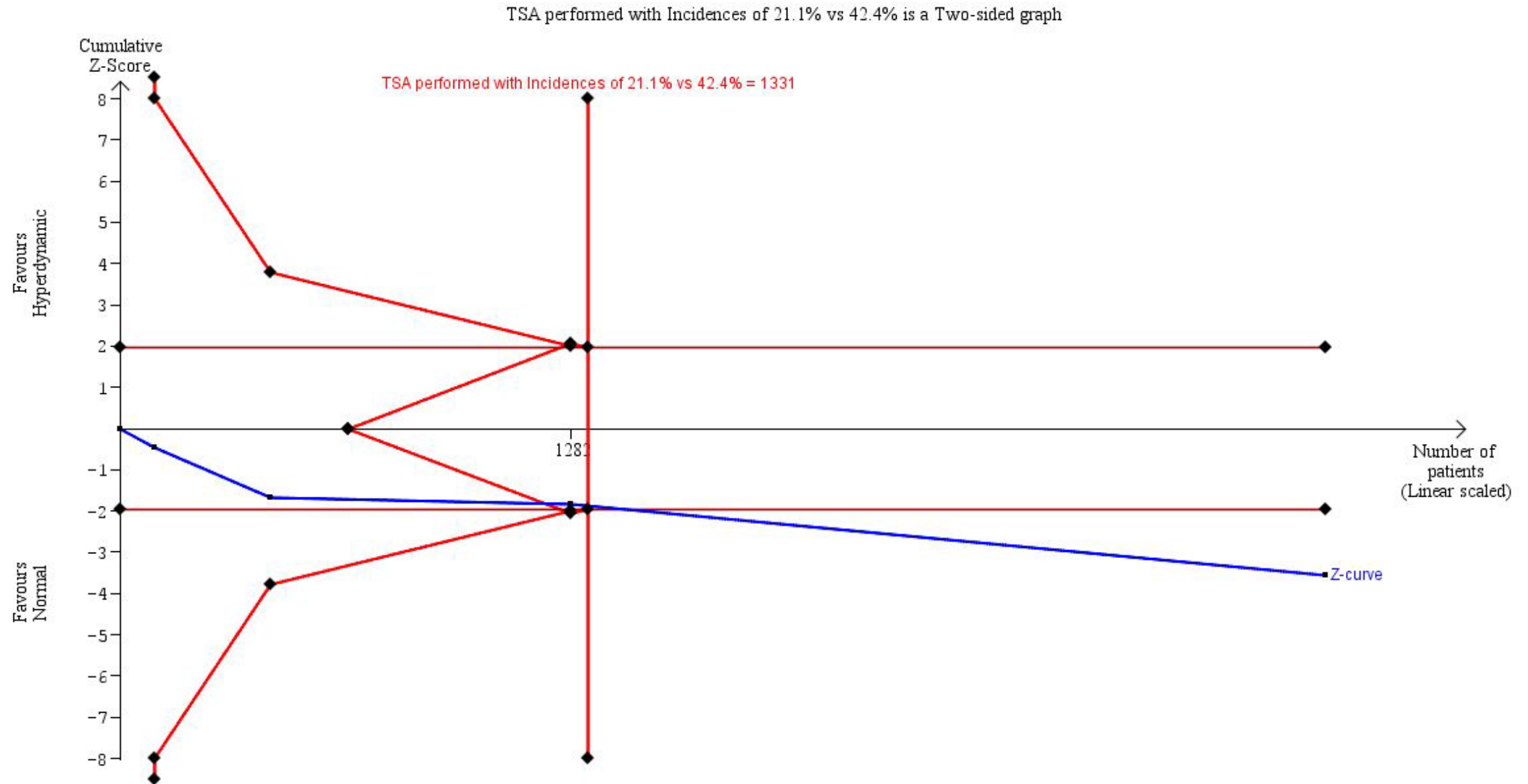

Supplement: Supplementary file 5 — Additional file 5. Trial sequential analysis. [file 13613_2024_1255_MOESM5_ESM.pdf]
