# Supplementary material for: Prevalence and prognosis of hyperdynamic left ventricular systolic function in septic patients: a systematic review and meta-analysis
Source: Ann Intensive Care. 2024 Feb 3;14:22. doi: 10.1186/s13613-024-01255-9 (PMC10838258; doi:10.1186/s13613-024-01255-9)
Supplement: Supplementary file 6 — Additional file 6: Table S1. Search strategy. [file 13613_2024_1255_MOESM6_ESM.docx]

**Table S1: Search strategy**

| Database | Date searched | Search items | Results found |
| --- | --- | --- | --- |
| MEDLINE (source, PubMed) | May 26^th^, 2022 | ((((Sepsis[mesh]) OR (Sepsis[tiab]) OR (septic shock [tiab]) OR (severe sepsis[tiab])) AND ("myocardial dysfunction" [tiab] or "left ventricular dysfunction" [tiab] or "systolic dysfunction" [tiab] or "cardiomyopathy" [tiab])) NOT ((animals[mh] NOT (animals[mh] AND humans[mh])))) | 1,223 |
| Cochrane Central Register of Controlled Trials | May 26^th^, 2022 | ((((Sepsis[mesh]) OR (Sepsis[tiab]) OR (septic shock [tiab]) OR (severe sepsis[tiab])) AND ("myocardial dysfunction" [tiab] or "left ventricular dysfunction" [tiab] or "systolic dysfunction" [tiab] or "cardiomyopathy" [tiab])) NOT ((animals[mh] NOT (animals[mh] AND humans[mh])))) | 285 |
| Embase | May 26^th^, 2022 | #1: ('septic shock'/exp OR 'septic shock') AND [humans]/lim  #2: ('severe sepsis'/exp OR 'severe sepsis') AND [humans]/lim  #3: ('sepsis'/exp OR 'sepsis') AND [humans]/lim  #4: ('myocardial dysfunction'/exp OR 'myocardial dysfunction') AND [humans]/lim  #5: ('left ventricular dysfunction'/exp OR 'left ventricular dysfunction') AND [humans]/lim  #6: ('systolic dysfunction'/exp OR 'systolic dysfunction') AND [humans]/lim  #7: ('cardiomyopathy'/exp OR 'cardiomyopathy') AND [humans]/lim  #1 OR #2 OR #3  #4 OR #5 OR #6 OR #7  #8 AND #9  #8 AND #9 AND ([adult]/lim OR [young adult]/lim OR [middle aged]/lim OR [aged]/lim OR [very elderly]/lim)  #11 AND [embase]/lim NOT ([embase]/lim AND [medline]/lim) | 1,094 |
