# Supplementary material for: Prevalence and prognosis of hyperdynamic left ventricular systolic function in septic patients: a systematic review and meta-analysis
Source: Ann Intensive Care. 2024 Feb 3;14:22. doi: 10.1186/s13613-024-01255-9 (PMC10838258; doi:10.1186/s13613-024-01255-9)
Supplement: Supplementary file 7 — Additional file 7: Table S2. Prevalence, heart rate, echocardiographic variables and Outcomes. [file 13613_2024_1255_MOESM7_ESM.docx]

**Table S2: Outcomes**

| **Prevalence** | | **Short-term mortality** | **Average E/e’** | **HR** | **LVEDD** |
| --- | --- | --- | --- | --- | --- |
| Dugar  2023 | Hyperdynamic (n=353) | 148/353 (41.9%)  470/1,792 (26.2%)  151/568 (26.6%)  106/305 (34.8%)  68/133 (51.1%) | 10.0 (7.7-12.6)  9.5 (7.4-12.2)  10.6 (8.0-14.0)  11.6 (9.4-15.7)  17.0 (10.9-23.7) | 96 (20)  90 (20)  92 (20)  94 (22)  101 (23) | 4.1 (3.5-4.5)  4.3 (3.8-4.8)  4.5 (4.1-5.1)  5.0 (4.4-5.5)  5.4 (4.6-6.2) |
|  | Normal (n=1,792)  Low normal (n=568)  Low (n=305)  Very low (n=133) |  |  |  |  |
| Chotalia  2022 | Hyperdynamic (n=175) | 103/175 (58.9%)  167/677 (24.7%)  55/162 (34.0%) | 8.8 (7.0-11.0)  8.6 (6.8-11.3)  10.1(7.6-12.4) | 95 (85-105)  86 (76-99)  88 (78-102) | 4.2 (3.8-4.6)  4.3 (4.0-4.7)  4.6 (4.2-5.2) |
|  | Normal (n=677)  Low (n=162) |  |  |  |  |
| Shin  2020 | Hyperdynamic (n=78) | 27/78 (34.6%)  63/252 (25.0%)  13/36 (36.1%) | 11.6 (5.8)  12.3 (5.2)  13.5 (4.5) | 93 (22)  91 (20)  98 (25) | 4.3 (0.7)  4.6 (0.6)  0.49 (0.6) |
|  | Normal (n=252)  Low (n=36) |  |  |  |  |
| Chang  2015 | Hyperdynamic (n=31) | 11/31 (35.5%)  21/69 (30.4%)  7/11 (63.6%) | -  -  - | -  -  - | -  -  - |
|  | Normal (n=69)  Low (n=11) |  |  |  |  |

HR: heart rate, LVEDD: left ventricular diastolic diameter
