# Supplementary material for: Prevalence and prognosis of hyperdynamic left ventricular systolic function in septic patients: a systematic review and meta-analysis
Source: Ann Intensive Care. 2024 Feb 3;14:22. doi: 10.1186/s13613-024-01255-9 (PMC10838258; doi:10.1186/s13613-024-01255-9)
Supplement: Supplementary file 8 — Additional file 8: Table S3. Newcastle–Ottawa Scale assessment of pooled studies. [file 13613_2024_1255_MOESM8_ESM.docx]

**Table S3: Newcastle-Ottawa Scale assessment of pooled studies.**

| **Study** | **Selection** | | | | **Comparability** | **Outcomes** | | | **Total** |
| --- | --- | --- | --- | --- | --- | --- | --- | --- | --- |
|  | Representativeness of exposed cohort | Selection of nonexposed cohort | Ascertainment of exposure | Outcome not present at the start of the study |  | Assessment of outcomes | Length of follow-up | Adequacy of follow-up |  |
| Dugar 2023 | * | * | * | * | -- | * | * | * | 7 |
| Chotalia 2022 | * | * | * | * | -- | * | * | * | 7 |
| Shin 2020 | * | * | * | * | -- | * | * | * | 7 |
| Chang 2015 | * | * | * | * | -- | * | * | * | 7 |

As we used the number of patients survived or died in those who with and without hyperdynamic LV fiunction, we did not use the adjusted number for comparability. Therefore, we rated 0 for comparability for each study.
