# Supplementary material for: Prevalence and prognosis of hyperdynamic left ventricular systolic function in septic patients: a systematic review and meta-analysis
Source: Ann Intensive Care. 2024 Feb 3;14:22. doi: 10.1186/s13613-024-01255-9 (PMC10838258; doi:10.1186/s13613-024-01255-9)
Supplement: Supplementary file 9 — Additional file 9: Table S4. GRADE (Grading of Recommendations, Assessment, Development, and Evaluations). [file 13613_2024_1255_MOESM9_ESM.docx]

**Table S4: GRADE (Grading of Recommendations, Assessment, Development, and Evaluations)**

| Prevalence of Prognosis of Hyperdynamic Left ventricular Systolic Function in Septic Patients: a Systematic Review and Meta-analysis | | | | | | | | | | | |
| --- | --- | --- | --- | --- | --- | --- | --- | --- | --- | --- | --- |
| Certainty assessment | | | | | | | **Summary of findings** | | | | |
| Participants (studies) Follow-up | Risk of bias | Inconsistency | Indirectness | Imprecision | Publication bias | Overall certainty of evidence | Study event rates (%) | | Relative effect (95% CI) | Anticipated absolute effects | |
|  |  |  |  |  |  |  | Hyperdynamic | Normal |  | Risk with hyperdynamic | RD with hyperdynamic (95% CI) |
| Prevalence | | | | | | | | | | | |
| 3,427 (4 studies) | not serious | not serious | not serious | serious^a^ | none | ⨁⨁◯◯ Low | 637/3,427 | 2,790/3,427 | --- | The prevalence was 182 per 1.000 | --- |
| Short-term mortality | | | | | | | | | | | |
| 3,427 (4 studies) | not serious | not serious | not serious | serious^a^ | none | ⨁⨁◯◯ Low | 270/637 | 590/2,790 | OR 2.37  (1.47 – 3.80) | RR 1.81  (1.40 – 2.34) | RD: 0.18  (0.06 – 0.30) |
| Average E/e’ | | | | | | | | | | | |
| 3,327 (3 studies) | not serious | not serious | not serious | serious^a^ | none | ⨁⨁◯◯ Low | --- | --- | SMD +0.03  (-0.09, +0.16) | --- | MD 0.17 higher  (-0.24 - +0.58) |
| Heart rate | | | | | | | | | | | |
| 3,327 (3 studies) | not serious | not serious | not serious | serious^a^ | none | ⨁⨁◯◯ Low | --- | --- | SMD +0.31  (+0.13, +0.49) | --- | MD 6.14/min higher  (+3.59 - +8.69) |
| Left ventricular diastolic diameter | | | | | | | | | | | |
| 3,327 (3 studies) | not serious | not serious | not serious | serious^a^ | none | ⨁⨁◯◯ Low | --- | --- | SMD -0.32  (-0.45, -0.18) | --- | MD 0.21cm smaller  (-0.33 - -0.09) |

**CI:** confidence interval; **RD:** risk difference, **MD:** mean difference; **OR:** odds ratio, **RR**: relative risk

#### Explanations

a. The sonographer experience and training could vary across the studies
